# Engaging children and young people on the potential role of artificial intelligence in medicine

**DOI:** 10.1038/s41390-022-02053-4

**Published:** 2022-04-07

**Authors:** Sheena Visram, Deirdre Leyden, Oceiah Annesley, Dauda Bappa, Neil J. Sebire

**Affiliations:** 1 Department of Computer Science | UCL Interaction Centre, University College London, London, UK; 2 DRIVE Centre, Great Ormond Street Hospital for Children, London, UK; 3 Young Persons Advisory Group (YPAG), Great Ormond Street Hospital for Children, London, UK

## Abstract

**Introduction:**

There is increasing interest in Artificial Intelligence (AI) and its application to medicine. Perceptions of AI are less well-known, notably amongst children and young people (CYP). This workshop investigates attitudes towards AI and its future applications in medicine and healthcare at a specialised paediatric hospital using practical design scenarios.

**Method:**

Twenty-one members of a Young Persons Advisory Group for research contributed to an engagement workshop to ascertain potential opportunities, apprehensions, and priorities.

**Results:**

When presented as a selection of practical design scenarios, we found that CYP were more open to some applications of AI in healthcare than others. Human-centeredness, governance and trust emerged as early themes, with empathy and safety considered as important when introducing AI to healthcare. Educational workshops with practical examples using AI to help, but not replace humans were suggested to address issues, build trust, and effectively communicate about AI.

**Conclusion:**

Whilst policy guidelines acknowledge the need to include children and young people to develop AI, this requires an enabling environment for human-centred AI involving children and young people with lived experiences of healthcare. Future research should focus on building consensus on enablers for an intelligent healthcare system designed for the next generation, which fundamentally, allows co-creation.

**Impact:**

- Children and young people (CYP) want to be included to share their insights about the development of research on the potential role of Artificial Intelligence (AI) in medicine and healthcare and are more open to some applications of AI than others.
- Whilst it is acknowledged that a research gap on involving and engaging CYP in developing AI policies exists, there is little in the way of pragmatic and practical guidance for healthcare staff on this topic.
- This requires research on enabling environments for ongoing digital cooperation to identify and prioritise unmet needs in the application and development of AI.

## Introduction

There is growing interest in the application of Artificial Intelligence (AI) to medicine. Initially described as exotic, expensive, and not of benefit to ordinary people,^1^ global interest within the field has increased exponentially.^2^ High-quality reviews of AI in healthcare have addressed its use, value, and trustworthiness.^3–6^ In children’s healthcare, parents prioritise quality/accuracy, privacy, shared decision making, convenience, cost, human element of care, and social justice when evaluating AI-driven technologies.^7^ However, whilst views of children and young people (CYP) can shape healthcare provision,^8–11^ few policy recommendations reflect their views and beliefs.^12^ This is particularly the case for CYP with tacit healthcare knowledge.

Great Ormond Street Hospital for Children (GOSH) is the largest paediatric centre in the UK and an international centre of excellence for many clinical specialties. As part of the hospital, the Digital Research, Innovation, and Virtual Environments (DRIVE) Hub aims to accelerate research and deployment of new technology including working with patients and families to optimise technologies such as AI. The Young Persons Advisory Group (YPAG) is a patient and public involvement group embedded at the hospital comprising CYP who are interested in improving health by advising on research, and forms part of a national network (Generation R). The Young Persons Advisory Group for research (YPAG) at GOSH are an established group of thirty-seven individuals (at the date of reporting), represented well across the age range, ethnic background, and gender.

Using a workshop entitled AI&me, we make a contribution to a gap in knowledge, by exploring the perspectives of CYP with lived experiences of healthcare including establishing priorities of GOSH YPAG in a Patient and Public Involvement and Engagement (PPEI) workshop on Healthcare AI. PPEI comprises mechanisms to connect with the public, engage with researchers on preliminary ideas for future studies and a key ingredient to achieve a shared responsibility for health^13–15^

## Method

A PPEI workshop was designed to examine the potential for AI applications in medicine and healthcare with CYP, involving patients and the public from the outset as part of scoping future research questions and design.^16^

In preparation for this workshop, several steps were taken to set the context to healthcare and reduce potential bias. Firstly, YPAG were specifically chosen as a working group who advise routinely on healthcare-related topics to hospitals. As part of information shared in advance of the session, the scope of the PPEI session was clearly presented to capture perceptions of the potential role of AI in medicine. Secondly, as part of the workshop design, the Director of the DRIVE Hub at the hospital, opened the session with an introduction to the digital trajectory at the hospital. The intention to use enabling technologies, data, and analytics to provide safer, smarter, and kinder care but the introduction did not depict the role of AI as either positive or negative, and instead framed the workshop as a mechanism to talk to CYP interested in AI in medicine, including about worries, and reflect on their feedback to shape future research questions and ongoing involvement. This was described in the introduction. Thirdly, the design scenarios were specifically contextualised to healthcare applications as part of the workshop planning, as were the questions which served as probes.

Findings were reported as early themes using conventional content analysis and although traditionally used for focus groups, the COREQ 32-point checklist was used to cross check for reflexivity, design, and analysis (included as a supplement).^17^ Although not a requirement for a PPEI workshop, it is intended to be used for future research involving focus groups and interviews and so forms an important part of research design.

### Sampling

Members of GOSH YPAG who expressed an interest in AI in medicine contributed to a PPEI workshop run virtually, lasting one hour to explore their perceptions on the potential for AI in medicine and healthcare. As part of this they rated levels of comfort with AI-related design scenarios in healthcare and discussed mechanisms to effectively engage with patients and families on AI’s future potential.

### Design

The virtual workshop was opened with a short introduction to the digital trajectory at the hospital, where the workshop was framed as an interactive workshop to generate new perspectives on technological development with CYP. The workshop comprised four sections:

- An Introduction to Artificial Intelligence and the Digital Trajectory at GOSH
- A visioning poll on how comfortable those attending feel about AI in different scenarios
- Probing further about levels of comfort on the role of AI in helping to make decisions and taking the lead on making decisions
- A probe to ask “As healthcare professionals, how can we talk better to our patients and their families about AI?”

This included nine design scenarios that were presented, with future applications to healthcare including: Virtual Reality visits to hospitals, cleaning robots, talking robots, chatbots to diagnose disease, self-driving vehicles, AI-powered nurses, 3D printed hearts and sensor technology to reduce overcrowding. These were developed from a recent survey of 2000 parents about their levels of comfort with AI.^18^ Effort was made to introduce each scenario in a balanced way, the fact that levels of comfort were polled encouraged perceived risks and downsides to be surfaced as part of the dialogue. Quantitative polling of scenarios was undertaken anonymously using a 10-point Likert scale. To collect comments and provide several ways for CYP to participate, a virtual chat function and an agile, Audience Response System (Mentimeter AB, Stockholm, Stockholms Lan, Sweden) were used since these are effective for encouraging participation in virtual learning environments.^19,20^

The workshop predominantly focused on healthcare applications of technologies intended to delight, inform, predict, automate, or diagnose/treat and probed further by asking about mechanisms that would better engage CYP and their families about AI in medicine. Comments were collected verbatim.

### Data analysis

Content analysis of the narrative responses and dialogue, involved coding and categorising text data collected during the PPEI workshop^21,22^ This involved data familiarisation, immersion and iterative identification of codes, concepts, phrases, and language. Open codes were collated under categories arising from the data, subcategories merged as an iterative process and findings summarised with supporting verbatim quotes. This was conducted using NVivo for Windows v.1.4.1 (QSR Inter-national, Melbourne, Australia). Recent studies have used content analysis to analyse narratives on perceptions of AI amongst adults in medicine.^23^ In this instance the goal of drawing categories or early themes from the data using conventional, inductive content analysis was to shape future research scope with CYP through subjective interpretation of verbal and text data. This is important as there is limited existing qualitative research regarding perceptions of AI in medicine and healthcare amongst CYP.

Due to the context-specific nature of this workshop to healthcare, and to AI applications in healthcare and medicine, the coding team allowed for extant influences on the thematic coding beyond healthcare. Whilst this is a limitation, it is believed to be of limited impact because of the clear focus and scope of the PPEI workshop to address the potential role of AI in medicine from the offset, and role of YPAG to routinely feedback on topics exclusive to healthcare.

## Results

Twenty-one YPAG members (aged 10–21 years) participated in the workshop, generating 128 unique comments across platforms. The language used by participants comprised words that described how AI made them feel (58 generalised occurrences that included “affect”, “care”, “compassion”, “consider”, “experience” and “fear”), AI was commonly referred to as a “robot” (18 incidents) and “creepy” on six occasions. Patients were commonly mentioned (18 occurrences) and generalised words relating to comfort (“assure” and “reassure”) were used twenty-six times. The comments were conversational, but several comments were structured as questions (*n* = 28, 22%) suggesting interest to understand more about AI.

### Design scenarios

**Fig. 1:**
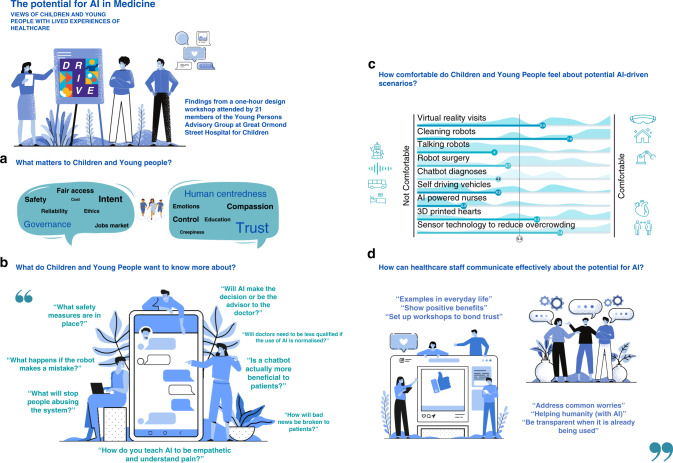
Twenty-one GOSH YPAG members (aged 10-21 years) participated in the one-hour workshop. **a** Human-centeredness, governance and trust emerged as major themes, with **b** empathy and safety considered as important when introducing AI to healthcare **c** A series of AI-enabled scenarios were scored by 21 CYP on a 10-point Likert scale for levels of comfort. Scores on aggregate were neutral for comfort (mean 5.3). Sensor technology to reduce overcrowding (M 7.4, SD 2.7), cleaning robots (M 7.9, SD 2.4), virtual reality visits (M 6.5, SD 2.8) and 3D printed organs (M 6.2, SD 3.5) were the most acceptable, whilst AI-powered nurses the least acceptable (M 2.4, SD 2.3). **d** Educational workshops with practical examples using AI to help, but not replace, humans were suggested to address issues, build trust, and effectively communicate about AI.

Of the nine design scenarios presented, sensor technology to reduce overcrowding (M 7.4, SD 2.7), cleaning robots (M 7.9, SD 2.4), virtual reality visits (M 6.5, SD 2.8) and 3D printed organs (M 6.2, SD 3.5) were the most accepted scenarios, whilst AI-powered nurses the least (M 2.4, SD 2.3; Fig. 1c).

### Emerging themes

Three themes emerged from the PPEI workshop: governance, human centredness, and trust, indicating potential aspects of AI in medicine that CYP may be interested to explore in further detail (Fig. 1).

### Governance

Safety and benefits formed the basis of a number of early inquiries about AI that stemmed from the participants. There was an interest that access to AI-enabled technologies was fair and available to all. Ensuring safety, security risks, and reliability was of particular interest, one participant asking, “What safety measures are in place?” another ‘What happens if the robot make(s) a mistake or the software breaks down?” expanding to ask “Would the robot get the benefit of the doubt?” More broadly, on ethical use of AI, one participant asked “How do you stop people abusing the system?”

As members of YPAG at a specialist paediatric hospital, a number of questions were raised about the role of AI for rare diseases, and potential benefits to challenges faced in healthcare, one participant asking “Will it speed up waiting times in A&E?” and on effectiveness, one participant asked “If a rare disease occurs, how will the robot know what to do as there is no specific treatment?” another “How do you train AI if someone develops a new illness?” and “Is an online chat bot actually more beneficial to patients?”

### Human centredness

The role of human-centred care in healthcare was another emergent theme with empathy, agency and power dynamics considered important. It was thought that AI would not take emotions into account, and this could have an impact on treatment, especially where mental health and wellbeing are considered. One participant asked “How do you teach AI to be empathetic and understand pain?” another “How would bad news be broken to patients?”

Agency and control over the use of AI was a pertinent topic, one participant reflecting: “I like the idea of AI looking at scans and in surgery, but definitely not for decision making or patient interaction,” another asking, “Would AI make the decision or be the advisor to the doctor?” and “Would doctors be able to overrule AI if they’re not happy with the decision/ course of action?”

Replacing humans was commonly associated to the impact on jobs, one participant expressing “I don’t like the idea of robots taking jobs” and another asked “What will happen to the doctors who are working now?” expanding to “will their jobs get replaced?” Another participant reflected on the potential impact on skillset and disparities between countries using AI and others that do not, asking “Will doctors need to be less qualified if the use of AI is normalised?” A popular remark anticipated the role of AI as supportive rather than to replace healthcare staff, one participant stating, “I will find it ok as long as it is just helping and does not replace humans.”

### Trust

The influence of movies, games, and science fiction on perceptions of AI was a popular topic. This was mentioned at the start of the workshop with first reflections of AI as creepy, this was revisited during the live polling and related to the scenario of ‘Talking robots with healthcare staff’, this led to comments about creepiness, one participant stating “AI is creepy if it acts like a human” and again at the close of the session reflecting that people cannot always trust one another, and when combined with the influence of how robots are depicted in films, this might make it difficult to encourage trust overall. Pop culture and science fiction, often depict robots as evil, one participant reflecting “I think we watch too many sci-fi movies,” another “that’s why I’m scared of robots.”

When asked “As healthcare professionals, how can we talk better to our patients and their families about AI?” of the 22 CYP attending, 13 CYP contributed comments via the Audience Response System, displayed as a rolling grid of live responses. These included educational workshops with reassurance and practical examples that use AI to help, but not replace humans to address common worries, build trust and to effectively communicate about AI. To cultivate trust, it was recommended that healthcare staff are transparent about its use, with clear explanations and examples of its use in everyday life, one participant recommended “being transparent when you are already using it e.g., when AI is used in conjunction with surgeons” with “success stories & when things go wrong & how it was resolved.”

Ethical considerations about who would make decisions and what might happen should something go wrong were considered. One participant stating, “make sure you address common worries instead of avoiding them when explaining AI.” Overall, participants were interested to engage on further discussions about AI, and a generational gap was identified, that considers young people more open to and comfortable with AI in general.

“YPAG members are keen to be involved, for our perspective and ideas, especially as AI is our future.”

## Discussion

This is the first PPEI workshop to our knowledge conducted virtually and as a group to engage with CYP with lived experiences of healthcare regarding perceptions of AI in medicine and healthcare. The findings of this PPEI workshop with 21 CYP, are intended to inform future research and have demonstrated that CYP are open-minded to feeding back their opinions on AI in medicine and healthcare and believe that this technology will change everyday life in fundamental ways but find it difficult to articulate their views on how AI should be developed in medicine. This is partly due to the breadth of applications and their impacts.

When presented as a selection of practical design scenarios, we found that CYP were more open to some applications of AI in healthcare than others. No application of AI received a vote of complete comfort, and the average score of comfort on a 10-point Likert scale was 5.3 across all nine scenarios. We also found that CYP value the role of human connectedness, trust, and governance. These results reinforce prior findings from a study on parental openness and concern towards AI-driven technologies in paediatric healthcare, where social justice and the human element of care are important to parents, who are positively influenced by quality, faith in technology and trust in health information systems.^7^ We find that CYP express higher levels of comfort with robot cleaners, virtual reality visits to hospital, and sensors to reduce overcrowding into and out of public spaces, which concur with prior findings from the Generation AI 2020, a global IEEE survey of millennial parents.^18^ There are also instances of divergent attitudes across generations with nearly half of millennial parents feeling comfortable leaving their child in the care of an AI powered virtual nurse during a hospital stay.^18^ By contrast we found lower levels of comfort were expressed by CYP towards AI-powered nurses and chatbots, where AI might behave like or replace the role of humans. For healthcare, this highlights a need to involve both the viewpoints of CYP as well as parents, especially where attitudes differ towards novel applications of AI intended to improve the provision of care.

CYP need to be educated about AI and encouraged to participate in its development including making AI explainable to CYP by including them in AI policy development cycles.^10–12^ Outside of healthcare, UNICEF recommends nine requirements for child-centred AI, including inclusion, safety, privacy, transparency, and the need to create an enabling environment to discover whether AI systems are designed for children and potential impacts.^10,11^ Whilst policies describe participatory research as a key element, such guidelines are not accompanied by practical recommendations and tools to enable such digital cooperation and implementation in environments such as hospitals, which this workshop draws attention to.

Whilst the emergent themes are limited to the constraints of the design of a single PPEI workshop, we do demonstrate that by creating an open, enabling environment, and using design scenarios to discuss potential applications, YPAG members were keen to participate, share opinions, outline concerns, and further develop their own understanding of AI. By including and involving CYP in this space, we have the potential to optimise AI to enhance future experiences of care. To achieve this shared aspiration requires collaboration, and where there are areas of disagreement or uncertainty, these need to be clearly identified.^24^ This involves creating an enabling environment for CYP-centred AI and involving CYP with lived experiences of healthcare in the process in ways that engage, inspire and empower.^25,26^

### Limitations

The Audience Response System, and chat function collected comments anonymously. Whilst this makes it difficult to attribute findings as a representative view rather than that of a small number, the workshop was facilitated to ensure that everybody attending had opportunity to contribute in a number of ways: verbally, by typed comments and polling on design scenarios for AI in healthcare. This offered several ways to contribute as CYP might choose to participate differently and this was intended to create an open and honest environment. This is particularly important as the workshop was conducted during a pandemic period when face to face interactions were readily replaced with virtual ones. One example that reflects the broad participation is the polling of the nine design scenarios. Of the twenty-one attending the session, 20 CYP participated in the live polling.

The PPEI workshop is intended to inform future research questions and spur debate on this topic. Whilst content analysis represents an appropriate analytic approach that is unobtrusive, nonreactive, and time-efficient when compared to methods such as ethnography, this PPEI workshop in its design and findings reported are limited in numerous ways, by the breadth of specific potential AI applications discussed, and by the depth of discussion achieved during a PPEI workshop conducted virtually over one-hour as a group. This extends to the perceptions of AI in everyday life as well as those within healthcare, illustrated by references to science fiction. Data saturation was not intended to be achieved; rather involvement as a mechanism to foster communication, capturing the voice and insights of CYP as part of an iterative process to shape future research. This is intended to instigate a dialogue, capture curiosity and shape research questions on artificial intelligence in medicine. Whilst the design of this PPEI workshop allowed for rapid, and well attended virtual participation to be achieved during pandemic period restrictions, subsequent research informed by this PPEI workshop would benefit from improved study design, rigour in qualitative methods applied, and include Supplementary data collection, in-depth interviews, to triangulate findings followed by consensus-building methods like a Delphi study, reported using evaluation tools like GRIPP2.^27^

## Conclusion

CYP want to be included to share their voice and insights about the development of research on the potential role of AI in medicine and healthcare. Whilst policy guidelines acknowledge the need to include CYP this ignores the infrastructure required to support ongoing digital cooperation. For AI in medicine, this requires an enabling environment for human-centred AI that involves CYP with lived experiences of healthcare and healthcare/AI professionals. With publication of the recent UK National Strategy for AI, w**e** recommend that future research should continue to iterate with CYP to shape an intelligent, empathetic, and inclusive healthcare system of tomorrow. Specifically, that future research should assess and evaluate the role of working groups like YPAG in creating an enabling environment to identify and prioritise unmet needs in the application and development of AI.

## Supplementary information

Supplement

Supplemental Materials

## Data Availability

The datasets generated and analysed may be available from the corresponding author upon reasonable request s.visram@ucl.ac.uk.
