# Supplementary material for: Engaging children and young people on the potential role of artificial intelligence in medicine
Source: Pediatr Res. 2022 Apr 7;93(2):440–4. doi: 10.1038/s41390-022-02053-4 (PMC9937917; doi:10.1038/s41390-022-02053-4)
Supplement: Supplementary file 1 — Supplement [file 41390_2022_2053_MOESM1_ESM.pdf]

Supplement. COREQ 32-point checklist for reporting on focus groups

No	Item	Description
**Domain 1: Research team and reflexivity**		
Personal Characteristics		
1.	Interviewer/facilitator	Facilitators (SV, NJS)
2.	Credentials	Undertaking PhD in Computer Science/ Human Computer Interaction (SV), Professor of Pathology and Chief Research and Informatics Officer (CRIO) (NJS)
3.	Occupation	PhD candidate and visiting researcher to GOSH (SV), Professor of Pathology, CRIO and Director of GOSH DRIVE (NJS)
4.	Gender	Female (SV), Male (NJS)
5.	Experience and training	Experienced - Undertaking PhD in Computer Science/ Human Computer Interaction (SV), Professor of Pathology and Chief Research and Informatics Officer (CRIO) (NJS)
Relationship with participants		
6.	Relationship established	Prototypes of new technologies are presented to YPAG in their role to provide feedback on research and so the participants were familiar with who the facilitators were.
7.	Participant knowledge of the interviewer	Participants knew the credentials of the facilitators and that SV is conducting research on human-centred aspects of technology adoption.
8.	Interviewer characteristics	The facilitators are interested in new technologies. This is an exploratory workshop and did not intend to prove or disprove a hypothesis, but instead garner perceptions of children and young people to shape future research.
**Domain 2: Design**		
Theoretical framework		

Continued on next page

9.	Methodological orientation and Theory	Descriptive statistics of quantitative polling of design scenarios involving AI, and Content analysis of 128 short comments or micronarratives.
Participant selection		
10.	Sampling	Purposive selection as members of YPAG
11.	Method of approach	Email invitation by Patient and Public Involvement Research Lead, NIHR GOSH Biomedical Research Centre
12.	Sample size	21
13.	Non-participation	0 – participation was voluntary for this engagement session
Setting		
14.	Setting of data collection	Virtually via a video conferencing platform with comments posted anonymously to an audience response system and in chat functions.
15.	Presence of non-participants	Patient and Public Involvement Research Lead, NIHR GOSH Biomedical Research Centre (1) , Biomedical Research Centre staff (1), Biomedical Research Centre staff as a note taker for field notes (1)
16.	Description of sample	Children and young people ages 10-21 years of age, typically with lived experiences of healthcare and members of YPAG, an advisory group that feeds back on research.
Data collection		
17.	Interview guide	The session was introduced with an agenda two months prior to the session and the questions were circulated to the team at GOSH DRIVE
18.	Repeat interviews	No
19.	Audio/visual recording	No
20.	Field notes	One independent observer took field notes during the session which were used to cross reference codes and emerging themes from the data
21.	Duration	One hour

Continued on next page

22.	Data saturation	This was an exploratory workshop and so data saturation was not anticipated
23.	Transcripts returned	Comments made in the chat function were made available to participants, and comments made anonymously on the audience response systems displayed as a rolling grid in real time during the session.
**Domain 3: analysis and findings**		
Data analysis		
24.	Number of data coders	1 (SV)
25.	Description of the coding tree	Yes, for each emerging theme (human centredness, governance and trust) Coding tree on NVivo (Figure 2)
26.	Derivation of themes	Derived inductively from the data
27.	Software	NVivo for Windows v.1.4.1 (QSR International, Melbourne, Australia)
28.	Participant checking	One participant was invited to cross check emerging codes and themes for accuracy and co-author the paper that presented preliminary findings
Reporting		
29.	Quotations presented	Yes, quotations were presented, the participants were not identified as comments were made anonymously. This was intended to encourage open and honest discussions
30.	Data and findings consistent	Yes
31.	Clarity of major themes	Yes, for each emerging theme (human centredness, governance and trust)
32.	Clarity of minor themes	Yes, for divergence in opinions and open coding
