# Supplementary material for: Engaging children and young people on the potential role of artificial intelligence in medicine
Source: Pediatr Res. 2022 Apr 7;93(2):440–4. doi: 10.1038/s41390-022-02053-4 (PMC9937917; doi:10.1038/s41390-022-02053-4)
Supplement: Supplementary file 2 — Supplemental Materials [file 41390_2022_2053_MOESM2_ESM.pdf]

Supplemental Materials

A) POLLING RESULTS

B) POLLING RESULTS AS THEY APPEAR ON THE AUDIENCE RESPONSE SYSTEM AS A GRID OF LIVE RESULTS

C) EXAMPLE OF FLOW OF CONTRIBUTIONS TO CHAT FROM SEVERAL PARTICIPANTS

D) RESPONSES AS THEY APPEAR ON THE AUDIENCE RESPONSE SYSTEM AS A GRID OF LIVE RESULTS

E) EXAMPLE OF FLOW OF CONTRIBUTIONS TO CHAT ON SAFETY CONSIDERATIONS

F) CONTRIBUTIONS ABOUT SCIENCE FICTION MADE AT THE BEGINNING AND CLOSE OF THE WORKSHOP

G) DEMOGRAPHICS ABOUT GOSH YPAG MEMBERSHIP AT THE TIME OF REPORTING

a) Polling results to nine AI-driven design scenarios

Date	Session	Voter	How comfortable do you feel with AI Agents?: Virtual reality visits	How comfortable do you feel with AI Agents?: Cleaning robots	How comfortable do you feel with AI Agents?: Talking robots	How comfortable do you feel with AI Agents?: Robot surgery	How comfortable do you feel with AI Agents?: Chatbot diagnoses	How comfortable do you feel with AI Agents?: Self driving vehicles	How comfortable do you feel with AI Agents?: AI powered nurses	How comfortable do you feel with AI Agents?: 3D printed hearts	How comfortable do you feel with AI Agents?: Sensor technology to reduce overcrowding
2020-11-28	1	**1**	4	5	3	8	5	7	5	8	9
2020-11-28	1	**2**									
2020-11-28	1	**3**	10	9	8	1	2	8	3	9	10
2020-11-28	1	**4**	8	8	4	2	1	2	0	2	4
2020-11-28	1	**5**	5	9	3	7	7	0	0	8	7
2020-11-28	1	**6**	10	10	10	9	5	10	8	8	8
2020-11-28	1	**7**	1	7	1	5	1	1	1	10	10
2020-11-28	1	**8**	10	10	10	5	10	5	5	1	5
2020-11-28	1	**9**	3	5	5	6	5	5	1	8	7
2020-11-28	1	**10**	7	9	2	6	7	8	3	9	8
2020-11-28	1	**11**	7	10	1	5	2	2	1	5	10
2020-11-28	1	**12**	5	10	5	7	4	7	2	10	10
2020-11-28	1	**13**	8	7	0	0	0	0	0	0	0
2020-11-28	1	**14**	10	5	0	0	3	2	0	7	5
2020-11-28	1	**15**	4	9	5	6	0	2	1	10	8
2020-11-28	1	**16**	2	1	6	0	3	2	1	5	7
2020-11-28	1	**17**	7	5	3	8	10	5	1	0	6
2020-11-28	1	**18**	7	9	6	8	6	4	5	8	9
2020-11-28	1	**19**	8	9	4	5	7	4	6	6	10
2020-11-28	1	**20**	10	10	2	6	2	7	3	10	10
2020-11-28	1	**21**	5	8	4	1	5	1	3	1	3
2020-11-28	1	**22**	5	10	3	3	3	7	1	5	9

B) POLLING RESULTS AS THEY APPEAR ON THE AUDIENCE RESPONSE SYSTEM AS A GRID OF LIVE RESULTS

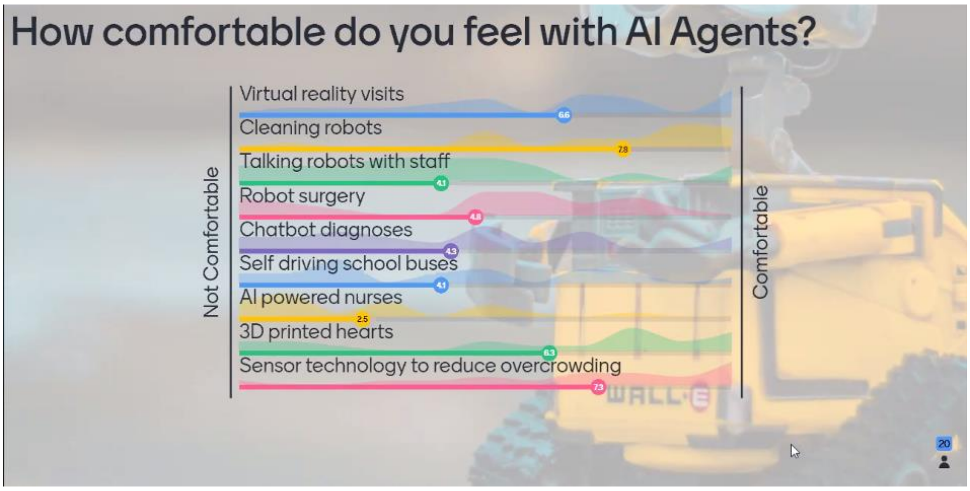

C) EXAMPLE CONTRIBUTIONS TO CHAT

13:56:05 From: What will happen to the doctors who are working now

13:56:16 From: Will their jobs get replaced?

13:57:02 From: Great questions YPAG - keep them coming :)

13:57:28 From: I like the idea of AI in looking at scans and in surgery, but definitely not for decision making or patient interaction

13:57:45 From: i agree with .....

13:57:49 From: I agree

13:57:51 From: how expensive will it be to manufacture the robots

13:58:08 From: I agree with ......, I think a HUD would be better than actual interaction, leaving all decision making to the human

D) RESPONSES AS THEY APPEAR ON THE AUDIENCE RESPONSE SYSTEM AS A GRID OF LIVE RESULTS

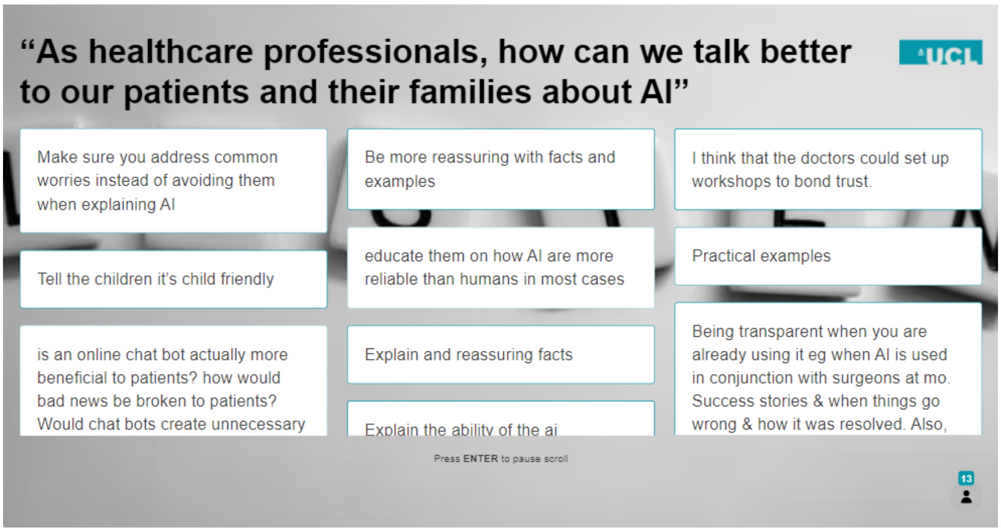

E) EXAMPLE OF CONTRIBUTIONS ON SAFETY CONSIDERATIONS

14:00:02 From: what happens if the robots make a big mistake or the software breaks down , would the hole healthcare system collapse?

14:00:34 From: Will doctors need to be less qualified if the use of AI is normalised?

14:00:46 From: there are ethical considerations.

14:07:57 From: Would doctors be able to over rule AI if they're not happy with the decision/ course of action?

14:08:11 From: how do you teach AI to be empathetic and understand pain

14:08:48 From: if a rare disease occurs, how will the robot know what to do as there is no specific treatment.

14:09:07 From: would the robot get the benifit of doubht

14:09:35 From: shouldn't the ai just be programed to do 1 thing

f) EXAMPLE CONTRIBUTIONS ON THE INFLUENCE OF SCIENCE FICTION

13:44:40 From: I agree creepy

13:44:49 From: the power of technology

14:27:42 From: Robots are particularly important in maintaining structure in everyday life but are difficult to emotionally attach with human (importance of nurses).

14:28:32 From: ai is creepy if it acts like a human

14:29:18 From: agreed I wish acted different so not like a human but like something else that's not creepy

14:34:03 From: a lot of people may be influenced by pop-culture and sci-fi regarding AI

14:34:49 From: again a thing that I think might have a problem is the fact that many people cant trust each, and for people to trust robots with also lots of flims and videos about them being evil or not very useful will probably make it very hard for people to try and get other people to trust them.

g) DEMOGRAPHICS ABOUT GOSH YPAG MEMBERSHIP AT THE TIME OF REPORTING

White	No.	Mixed	No.	Asian or Asian British	No.	Black or Black British	No.	Chinese or other ethnic group	No.
British	11	White and Black Caribbean	1	Indian	2	African	5	Chinese	2
Other	3	White and Asian	4	Any other Asian (Afghan)	2	Caribbean	1		
Irish	1	Other	2	Any other Asian	1				
				Bangladeshi	2				

Age	Number
11	1
12	3
13	2
14	3
15	3
16	5
17	6
18	6
19	2
20	2
21	3
	37
Gender breakdown	
11 male/26 female	
